# Performance of Pediatric Risk of Mortality III and Pediatric Index of Mortality III Scores in Tertiary Pediatric Intensive Unit in Saudi Arabia

**DOI:** 10.3389/fped.2022.926686

**Published:** 2022-07-07

**Authors:** Ahmed S. Alkhalifah, Abdulaziz AlSoqati, Jihad Zahraa

**Affiliations:** ^1^Qatif Central Hospital, Al-Qatif, Saudi Arabia; ^2^King Fahad Medical City, Riyadh, Saudi Arabia

**Keywords:** Pediatric Risk of Mortality score, Pediatric Index of Mortality, mortality, pediatric intensive care unit, discrimination, calibration

## Abstract

**Objective:**

To assess the performance of the Pediatric Risk of Mortality III (PRISM III) and Pediatric Index of Mortality III (PIM III) indices in a tertiary pediatric intensive care unit (PICU) in Saudi Arabia and to identify the factors affecting the observed performance.

**Design:**

Retrospective, single-center study using data collected from the Virtual Pediatric Systems web-based database.

**Setting:**

King Fahad Medical City PICU, Saudi Arabia.

**Patients:**

All pediatric patients <14 years of age admitted between 1 January 2015, and 31 December 2019.

**Interventions:**

Comparison of PRISM III and PIM III performances in predicting mortality across different age groups, disease categories, and resuscitation decision statuses.

**Measurements:**

Normality of distribution was assessed using the Kolmogorov–Smirnov and Shapiro–Wilk tests. Patient characteristics were compared between survivors and non-survivors. The medians and ranges were calculated for continuous data, whereas frequencies and percentages were used for nominal data. The Mann–Whitney U test, Kruskal–Wallis test, and Chi-square test were used to compare the characteristics of survivors and non-survivors.

**Main Results:**

There was a significant difference between the predicted mortality and observed mortality in both the PRISM III and PIM III. Better discrimination was found after excluding do-not-resuscitate (DNR) patients. The worst calibration and discrimination were recorded for infants <12 months of age. The PRISM III performed significantly better in patients with metabolic/genetic and central nervous system illnesses. Non-DNR patients had a lower standardized mortality rate using the PRISM III and PIM III. The PRISM III and PIM III indices performed better in patients who died within the first week of admission.

**Conclusion:**

These models had sufficient discrimination ability and poor calibration. Since they were designed for particular patient characteristics and PICUs, further testing in different environments is necessary before utilization for planning and assessing performance. Alternatively, new models could be developed which are suitable for local PICUs.

## Introduction

Critically ill pediatric patients display significant variability in illness severity, disease course, and outcomes (1). This variability is usually multifactorial and can be related to the patient’s intrinsic factors, nature or stage of illness, available resources, therapeutic approach, and other factors (2). Hence, multiple pediatric intensive care unit (PICU) outcome indices were developed and used for prognostication, resource allocation, and performance comparison with other PICUs for quality improvement purposes (3–5). These tools are designed to predict an individual patient’s outcome based on a constellation of factors available at admission, including patient characteristics, severity of illness parameters, and laboratory workup (6, 7).

The Pediatric Risk of Mortality (PRISM) is a common physiologically based scoring system used to predict mortality in critically ill children. It was first developed in North American PICUs in 1988 by Pollack et al. and then updated to the Pediatric Risk of Mortality III (PRISM III) (7, 8). Additionally, the Pediatric Index of Mortality (PIM) is another widely used scoring system initially created by Shann et al. and modified to the Pediatric Index of Mortality II then III (PIM III) (6, 9). Both scoring tools are used for mortality prediction and quality control in several PICUs worldwide (6, 7). With the improvement of the delivery of advanced care and outcomes in PICUs, these scoring tools have shown variable mortality predictions over the years, and have been modified mainly to improve their prediction capacity (6, 10, 11).

Because the PRISM III and PIM III are widely used worldwide, they can be used as quality indicators to benchmark and compare outcomes and to guide PICUs in monitoring and improving their performance (4, 12–14). However, indices may overestimate or underestimate patient mortality if applied to different patient groups or populations. To the best of our knowledge, there have been no similar comparative studies in our population. This study aimed to assess the performance of the PRISM III and PIM III tools in the King Fahad Medical City (KFMC) PICU and to identify the factors that may have led to the observed performance differences.

## Materials and Methods

### Study Design and Settings

This retrospective study used the Virtual Pediatric Systems (VPS) web-based database (Los Angeles, CA, United States^1^) in which the KFMC PICU has participated in since 2014. The data were entered into the VPS by a trained and licensed medical record officer and retrieved and reviewed by the researchers (pediatric intensive care physicians). The data collected included baseline characteristics, patient origin, diagnosis at admission to the PICU, comorbidities (including hospital-acquired, device-related infections, advanced respiratory support and procedures), all variables needed to calculate the PRISM and PIM, and mortality status. Ethical approval to anonymously analyze the data was obtained from the KFMC Institutional Review Board (approval number: FWA00018774).

The PICU at the KFMC is a multidisciplinary unit in a tertiary hospital. It provides specialized care to patients with complex medical and surgical diseases, not including acute trauma or solid organ transplantation, and serves as a referral center for other hospitals in the region.

All pediatric patients <14 years of age admitted to the KFMC PICU between 1 January 2015 and 31 December 2019, were included in this study. Neonates and preterm patients <44 weeks of gestational age with neonatal diseases of age admitted to other units not included in the VPS database were excluded from our study. Children older than 14 years of age were admitted to the adult critical care unit per the Saudi ministry of health regulations and thus not included in this study.

Patients transferred to another hospital or unit and those still admitted to the PICU at the end of the study period were excluded because mortality status could not be determined. In addition, patients with incomplete data to calculate the PIM III and PRISM III indices were also excluded.

### Statistical Analyses

After data collection, IBM SPSS Statistics for Windows (version 25.0; IBM Corp., Armonk, NY, United States) was used for the statistical analyses.

Kolmogorov–Smirnov and Shapiro–Wilk tests were used to assess the normality of the distribution. Patient characteristics were compared between survivors and non-survivors. The medians and ranges were calculated for continuous numerical data, whereas frequencies and percentages were used for nominal data. The Mann–Whitney U test, Kruskal–Wallis test, and Chi-square test were used to compare the characteristics of survivors and non-survivors, and a *P*-value < 0.05 was considered statistically significant.

The performance of the PRISM III and PIM III scores was assessed by their discriminatory power and calibration in all patients, and subgroups were categorized by age, diagnosed disease at admission, and do-not-resuscitate (DNR) status. Discriminatory power (i.e., the ability to predict survival and death at admission for each patient) was assessed by calculating the area under the receiver operating characteristic curve (AUC-ROC). Simultaneously, the standardized mortality ratio (SMR) and 95% confidence interval (CI) were analyzed. The SMR was calculated using the mid-P test. The AUC-ROCs and SMRs with corresponding 95% CIs were calculated for each age subgroup and diagnostic category.

The Hosmer–Lemeshow goodness-of-fit test was used to evaluate the calibration of the scoring system, which refers to the level of agreement between individual probabilities, including predicted and observed mortality. A value of P > 0.05 was accepted as good calibration.

## Results

**FIGURE 1:**
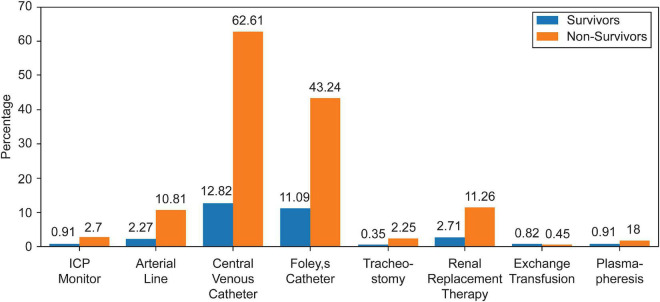
Procedures proportion.

**FIGURE 2:**
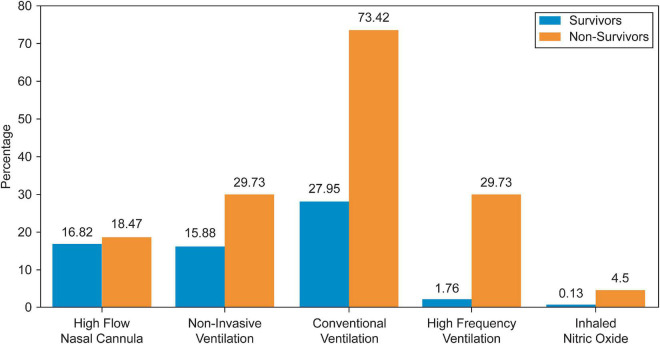
Respiratory support proportion.

A total of 4,019 admissions, corresponding to 2,620 different patients, were recorded during the study period. In 50 (1.2%) admissions, the patients were transferred to another PICU and were excluded, while 573 (14.3%) admissions, of which 95.5% were survivors, were excluded for missing data needed to calculate the indices. Thus, 3,396 admissions records were analyzed. Baseline patient characteristics are summarized in Table 1. The most frequent primary cause of admission was respiratory disease (30.3%), followed by infection-related illnesses (16.2%). Approximately 14.8% of the admitted patients had previous PICU admissions. More than a quarter (34.5%) of the included patients were admitted from the emergency department, 32.5% from the inpatient ward, and 28.8% postoperatively. When comparing survivors with non-survivors, non-survivors had significantly more comorbidities, hospital-acquired infections, and device-related infections than survivors. All patients with DNR status were in the non-survivor group, representing approximately 50% of all PICU mortalities. In addition, non-survivors required more procedures and advanced respiratory support during their PICU admission, as summarized in Table 2 and Figures 1, 2.

**TABLE 1:** Characteristics of the study population according to mortality.

	Mortality status		*P*-value
	Survivors	Non-survivors	
Number of patients	*n* = 3,174	*n* = 222	
PRISM III probability of death (%)			
Median (range)	0.51 (0.02-79.9)	3.42 (0.1-99.5)	<0.001
PIM III probability of death (%)			
Median (range)	0.72 (0.03-59.2)	3.14 (0.1-97.6)	<0.001
Patient-related factors			
Age (in months)			
Median (range)	35.15 (0.5-277.6)	21 (0.6-291)	<0.001
Height (cm)			
Median (range)	87 (6.7-185)	76 (35-163)	<0.001
Weight (kg)			
Median (range)	11.3 (1.5-98.4)	8 (1.6-72)	<0.001
By age group, *N* (%)			<0.001
≤12 M	897 (28.26%)	94 (42.34%)	
>12 to ≤60 M	1134 (35.73%)	71 (31.98%)	
>60 to ≤120 M	692 (21.80%)	32 (14.41%)	
>120 M	451 (14.21%)	25 (11.26%)	
Sex, *N* (%)			0.429
Male	1731 (54.54%)	115 (51.80%)	
Female	1443 (45.46%)	107 (48.20%)	
Diagnosis, *N* (%)			
Respiratory diseases	956 (30.12%)	74 (33.33%)	0.314
Infections	489 (15.41%)	62 (27.93%)	<0.001
Central nervous system diseases	509 (16.04%)	27 (12.16%)	0.126
Malignancies	366 (11.53%)	21 (9.46%)	0.348
Cardiovascular diseases	47 (1.48%)	7 (3.15%)	0.054
Immunological diseases	13 (0.41%)	9 (4.05%)	<0.001
Renal diseases	110 (3.47%)	6 (2.70%)	0.545
Metabolic/genetic diseases	131 (4.13%)	7 (3.15%)	0.477
Hematologic diseases	41 (1.29%)	1 (0.45%)	0.273
Endocrine diseases	158 (4.98%)	1 (0.45%)	0.002
Gastrointestinal diseases	170 (5.36%)	2 (0.90%)	0.003
Others	184 (5.80%)	5 (2.25%)	0.026
Post-operative admission, *N* (%)	960 (30.25%)	17 (7.66%)	<0.001
Previous ICU admission, *N* (%)	471 (14.84%)	49 (22.07%)	0.004
Patient origin, *N* (%)			<0.001
Ward	984 (31.00%)	120 (54.05%)	
Emergency	1103 (34.75%)	67 (30.18%)	
Operating room	960 (30.25%)	17 (7.66%)	
Another hospital ICU	86(2.71%)	18(8.11%)	
Other	41(1.3%)	0(0.00%)	
Comorbidities			
Median (range)	2(0−19)	3(0−18)	<0.001
Length of stay in hours			
Median (range)	64(1−5,632)	272(1−3,419)	<0.001
DNR status, *N* (%)	0(0.0%)	110(49.55%)	<0.001

*DNR, do-not-resuscitate; ICU, intensive care unit; PIM, Pediatric Index of Mortality; PRISM, Pediatric Risk of Mortality.*

**TABLE 2:** Complications during pediatric intensive care unit admission according to mortality.

	Mortality status		
	Survivors	Non-survivors	*P*-value
Number of patients	*n* = 3,174	*n* = 222	
VAP*^a^*, *N* (%)	12 (0.38%)	3 (1.35%)	0.035
CLABSI*^b^*, *N* (%)	34 (1.07%)	11 (4.95%)	<0.001
CAUTI*^c^*, *N* (%)	9 (0.28%)	2 (0.90%)	0.118
HAP*^d^*, *N* (%)	10 (0.32%)	5 (2.25%)	<0.001

*^a^Ventilator-associated pneumonia.*

*^b^Central line-associated bloodstream infection.*

*^c^Catheter-associated urinary tract infections.*

*^d^Hospital acquired pneumonia.*

### Pediatric Risk of Mortality III and Pediatric Index of Mortality III Performances

**FIGURE 3:**
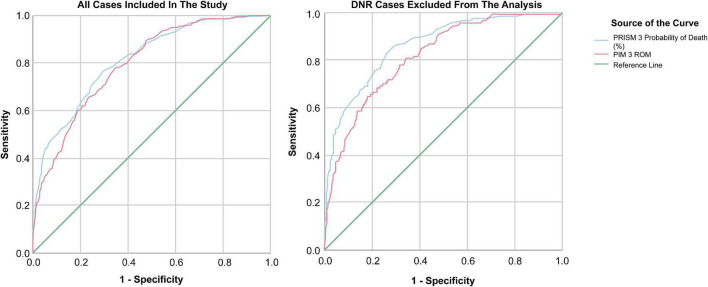
Mortality indices AUC-ROC.

The predicted mortality rates for the PRISM III and PIM III were 2.5 and 2.38%, respectively, for all patients included in the study. The observed mortality (6.54%, 222 deaths) was more than double that predicted by the indices, with a significant difference of *P* < 0.001. By further subcategorizing the patients according to the predicted risk of mortality, both the PRISM III and PIM III showed a statistically significant difference between observed and predicted mortalities in most of the deciles apart from lower-risk cases, as demonstrated in Tables 3, 4 (PRISM III χ^2^, 435.44 and PIM III χ^2^, 534.15; 5 degrees of freedom; *P* < 0.001). The AUC-ROC for both the PRISM III and PIM III was 0.81 (95% CI, 0.79–0.84; *P* < 0.001) and 0.80 (95% CI, 0.77–0.82; *P* < 0.001), respectively, when all patients were included. After excluding the patients with DNR status, the discrimination improved to 0.87 (95% CI, 0.84–0.90; *P* < 0.001) and 0.82 (95% CI, 0.79–0.86; *P* < 0.001), respectively as seen in Figure 3.

**TABLE 3:** Mortality observed and predicted per category of predicted risk for the Pediatric Risk of Mortality III*.

Categories of risk predicted by the PRISM III	Number of patients in category of predicted risk	Observed mortality, *n* (%)	Mortality predicted by the PRISM III, *n* (%)	*P*-value
0.00–0.19%	624	3 (0.48%)	0.85 (0.14%)	0.020
0.20–0.30%	417	4 (0.96%)	1.17 (0.28%)	0.009
0.31– 0.49%	427	12 (2.81%)	1.72 (0.40%)	<0.001
0.50–0.66%	478	18 (3.77%)	2.74 (0.57%)	<0.001
0.67–1.3%	513	29 (5.65%)	4.93 (0.96%)	<0.001
1.31–3.25%	490	44 (8.98%)	10.24 (2.09%)	<0.001
3.26–100%	447	112 (25.06%)	63.31 (14.16%)	<0.001
Total	3,396	222 (6.54%)	84.96 (2.50%)	<0.001

*χ^2^: 435.44; 5 degrees of freedom; P < 0.001. PRISM, Pediatric Risk of Mortality.

**TABLE 4:** Mortality observed and predicted per category of predicted risk for the Pediatric Index of Mortality III*.

Categories of risk predicted by the PIM III	Number of patients in categories of predicted risk	Observed mortality, *n* (%)	Mortality predicted by the PIM III, *n* (%)	*P*-value
0.00–0.22%	581	3 (0.52%)	0.84 (0.14%)	0.018
0.23–0.38%	401	2 (0.50%)	1.16 (0.29%)	0.435
0.39–0.69%	501	10 (2.00%)	2.69 (0.54%)	<0.001
0.70–0.84%	466	28 (6.01%)	3.54 (0.76%)	<0.001
0.85–1.22%	480	31 (6.46%)	4.78 (1.00%)	<0.001
1.23–3.51%	483	51 (10.56%)	10.42 (2.16%)	<0.001
3.54–100%	484	97 (20.04%)	57.44 (11.87%)	<0.001
Total	3,396	222 (6.54%)	80.85 (2.38%)	<0.001

*χ^2^: 534.15; 5 degrees of freedom; P < 0.001. PIM, Pediatric Index of Mortality.

### Indices Performance by Age Subgroups

Discrimination assessments for both indices showed variable accuracies across age groups. There was a significant difference in the mortality predictions across all age groups for the PRISM III and PIM III indices, indicating poor calibration. PRISM III’s best performance was found in children between 60 and 120-months old, with an AUC-ROC of 0.87 (95% CI, 0.82–0.93; *P* < 0.001). On the other hand, the PIM III was better in older patients but worse than the PRISM III in most age groups (Table 5).

**TABLE 5:** Performance per age group.

			PRISM III			PIM III		
Group	Number of patients	Observed mortality, *N* (%)	Standardized mortality ratio (95% CI)	ROC curve		Standardized mortality ratio (95% CI)	ROC curve	
				AUC (95% CI)	*P*-value		AUC (95% CI)	*P*-value
≤12M	991	94 (9.49%)	3.96 (3.16–4.76)	0.79 (0.74–0.84)	<0.001	3.42 (2.73–4.12)	0.76 (0.71–0.81)	<0.001
>12 to≤60 M	1,205	71 (5.89%)	2.27 (2.00–2.54)	0.81 (0.76–0.87)	<0.001	2.34 (2.06–2.62)	0.81 (0.75–0.86)	<0.001
>60 to ≤120 M	724	32 (4.42%)	2.23 (1.84–2.62)	0.87 (0.82–0.93)	<0.001	2.19 (1.80–2.57)	0.83 (0.76–0.89)	<0.001
>120 M	476	25 (5.25%)	1.61 (1.29–1.93)	0.81 (0.73–0.90)	<0.001	2.96 (2.37–3.56)	0.82 (0.76–0.88)	<0.001
Total	3,396	222 (6.54%)	2.61 (2.44–2.79)	0.81 (0.79–0.84)	<0.001	2.75 (2.56–2.93)	0.80 (0.78–0.82)	<0.001

*The P-value corresponding to χ2 was calculated as (observed deaths − expected deaths)^2^/expected deaths with one degree of freedom. AUC, Area Under the Curve; CI, confidence interval; PIM, Pediatric Index of Mortality; PRISM, Pediatric Risk of Mortality; ROC, receiver operating characteristic.*

The worst calibration and discrimination were recorded for infants <12 months of age in which the calculated SMR for the PRISM III and PIM III was 3.96 (95% CI, 3.16–4.76) and 3.42 (95% CI, 2.73–4.12), respectively. This age group had more patients admitted with infectious (254 patients; *P* < 0.001) and metabolic/genetic (69; *P* < 0.001), and immunological (18; *P* < 0.001) diseases than other age groups.

### Indices Performance by Disease Subgroups

The largest group (30.1%) of patients included in this study were admitted with respiratory-related illnesses, followed by those admitted with infections (16.2%) and neurological illnesses (15.8%). Mortality was the highest among patients admitted with immunological illnesses (40.9% mortality, 9/22), followed by cardiovascular (13%, 7/54) and infection-related illnesses (11.25%, 62/551). The PRISM III showed better overall discrimination in predicting mortality than the PIM III across different disease subcategories. The PRISM III performed significantly better in patients admitted with metabolic/genetic and central nervous system illnesses, with an AUC-ROC of 0.93 (95% CI, 0.87–0.99) and 0.90 (95% CI, 0.85–0.94), respectively. The PIM III performance was less discriminative across most disease subcategories. Both indices showed good agreement in patients with gastrointestinal, endocrine, and hematological illnesses.

The highest SMR for the PRISM III was recorded in patients admitted with immunological diseases (4.27; 95% CI, 1.48–7.05) and malignancies (4.74; 95% CI, 2.71–6.77), while for the PIM III, the highest was recorded in patients admitted with infectious diseases (4.01; 95% CI, 3.01–5.01). A detailed analysis of both indices is provided in Table 6. Patients with immunological and infectious diseases were found to have more comorbidities (median: 3; *P* < 0.001), while the lowest albumin (median: 2.31 g/dL, range: 1.5–3.6; *P* < 0.001) and hemoglobin levels (median: 7.90 g/dL, range: 6–11.1; *P* < 0.001) were recorded in patients admitted with immunological diseases.

**TABLE 6:** Performance per disease category.

	PRISM III			PIM III		
	SMR (95% CI)	ROC curve		SMR (95% CI)	ROC curve	
		AUC (95% CI)	*P*-value		AUC (95% CI)	*P*-value
All patients	2.61 (2.27–2.96)	0.81 (0.79–0.84)	<0.001	2.75 (2.56–2.93)	0.80 (0.77–0.82)	<0.001
All excluding DNR	1.52 (1.24–1.80)	0.87 (0.84–0.90)	<0.001	1.64 (1.49–1.80)	0.82 (0.79–0.86)	<0.001
**Disease group**						
Immunological	4.27 (1.48–7.05)	0.79 (0.59–0.98)	0.025	2.82 (0.98–4.66)	0.69 (0.46–0.93)	0.133
Metabolic/genetic	2.72 (0.71–4.74)	0.93 (0.87–0.99)	<0.001	1.97 (0.51–3.42)	0.71 (0.53–0.90)	0.057
Renal	1.28 (0.26–2.30)	0.86 (0.67–1.00)	0.003	2.90 (0.58–5.22)	0.78 (0.59–0.96)	0.023
Endocrine	2.44 (–2.34–7.22)	0.93 (0.90–0.97)	0.136	0.95 (–0.91–2.82)	0.99 (0.98–1.00)	0.089
CNS disease	2.80 (1.75–3.86)	0.90 (0.85–0.94)	<0.001	2.95 (1.84–4.07)	0.88 (0.82–0.95)	<0.001
Cardiovascular disease	1.97 (0.51–3.42)	0.80 (0.65–0.95)	0.011	2.27 (0.59–3.94)	0.58 (0.41–0.75)	0.511
GI/hepatic	1.04 (–0.40–2.47)	0.86 (0.66–1.00)	0.081	1.06 (–0.41–2.54)	0.92 (0.83–1.00)	0.041
Hematological	1.52 (–1.45–4.48)	1.00 (1.00–1.00)	0.091	2.04 (–1.96–6.04)	0.93 (0.85–1.00)	0.149
Infections	2.50 (1.88–3.13)	0.77 (0.71–0.84)	<0.001	4.01 (3.01–5.01)	0.74 (0.68–0.80)	<0.001
Respiratory	2.75 (2.13–3.38)	0.72 (0.66–0.79)	<0.001	2.68 (2.07–3.29)	0.85 (0.77–0.93)	<0.001
Malignancies	4.74 (2.71–6.77)	0.84 (0.76–0.92)	<0.001	2.45 (1.40–3.50)	0.82 (0.63–1.00)	0.016
Other	1.51 (0.19–2.83)	0.86 (0.70–1.00)	0.006	1.06 (0.13–1.99)	0.73 (0.67–0.78)	<0.001

*The P-value corresponding to χ2 was calculated as (observed deaths − expected deaths)^2^/expected deaths with one degree of freedom. AUC, Area Under the Curve; CI, confidence interval; CNS, central nervous system; DNR, do-not-resuscitate; PIM, Pediatric Index of Mortality; PRISM, Pediatric Risk of Mortality; ROC, receiver operating characteristic, SMR, standardized mortality ratio.*

### Non-survivors’ Analysis and Do-Not-Resuscitate Status

Non-survivors were further subcategorized according to DNR status and length of stay, as summarized in Table 7. Of the non-survivors, 50% signed DNR after their admission to the PICU. Almost half of the non-DNR mortalities occurred after 1 week of admission, while the majority (73%) of DNR mortalities occurred after 7 days. Patients who died within the first week of admission had significantly higher PRISM III (median, 11.9; range, 0.1–99.5; *P* = 0.01) and PIM III (median: 4.6, range: 0.4–93; *P* = 0.019) scores. The PRISM III and PIM III performed better in patients who died within the first week of admission (AUC-ROC of 0.90 and 0.86, respectively) than in patients who died after 7 days of admission (AUC-ROC of 0.63 and 0.64, respectively). Patients who died 1 week after admission had significantly more hospital-acquired infections. DNR patients who died after 1 week had more respiratory and neurological-related illnesses (40.7 and 22.2%, respectively; *P* = 0.005) than those in the other groups. There were no significant differences in the demographic data between the groups.

**TABLE 7:** Characteristics of the non-survivors according to do-not-resuscitate status.

	Non-DNR		DNR		
	0–7 days	>7 days	0–7 days	>7 days	*P*-value
Number of patients	55	57	29	81	
PIM III probability of death (%)					
Median (range)	4.9 (0.4–79)	3.23 (0.4–67)	3.51 (0.4–93)	2 (0.1–98)	0.012
PRISM III probability of death (%)					
Median (range)	14.2 (0.2–100)	2.8 (0.3–63)	5.3 (0.1–94)	1.6 (0.3–98)	<0.001
Comorbidities					
Median (range)	5.15 (±2.93)	5.44 (±2.48)	4.00 (±2.46)	4.49 (±2.82)	0.152
Hospital-acquired infections, *N* (%)	1 (1.82%)	9 (15.79%)	0 (0%)	10 (12.35%)	0.014

*DNR, do-not-resuscitate; PIM, Pediatric Index of Mortality; PRISM, Pediatric Risk of Mortality.*

## Discussion

This study was conducted to validate the performance of the PRISM III and PIM III indices in patients admitted to a tertiary health care facility in Riyadh, Saudi Arabia. The PRISM III generally showed better discrimination between survivors and non-survivors than the PIM III across all age groups. The lowest performance of both indices was observed in those ≤12 months of age. The PRISMS III performed less well in patients with respiratory diseases, whereas the PIM III showed less discrimination in patients with cardiovascular, immunological, and metabolic/genetic diseases. In contrast, PRISM III had the worst calibration in patients with immunological and malignancy-related diseases, whereas PIM III’s worst calibration was demonstrated in patients with infectious diseases. Similar observations were reported by Malhotra et al. in their study of comparable population groups (15). A number of studies conducted in different countries, including India and Austria, compared the performance of different indices and showed variable results between the indices in the populations studied (16–18). In 2021, Shen and Jiang reported a good overall but variable performance for mortality prediction in the PICU across studies included in their meta-analysis (19). The variations in calibration and discrimination between the PRISM III and PIM III across disease groups could be due to the difference in the parameters used to calculate each score.

The performance of the PRISM III in this study was weaker compared with that in another study performed in a tertiary medical center in Saudi Arabia, by Albuali et al. (20). Their study showed a PRISM III AUC-ROC of 0.955 (95% CI, 0.924–0.986) and a significantly higher PRISM III score in non-survivors. In their study, the sample size was much smaller and the proportions of metabolic, genetic, and immune-deficient patients were not mentioned, making real comparison inaccurate. To the best of our knowledge, no other studies have assessed the performance of the PRISM III or PIM III for similar populations and medical infrastructures. Other studies on PIM III performance conducted in different geographical areas and populations have shown results comparable to ours. One study conducted in another Middle Eastern country showed an AUC-ROC of 0.78 (95% CI, 0.69–0.87), while a multicenter study by Arias López et al. showed an AUC-ROC of 0.84 (95% CI, 0.82–0.87) and 0.82 (95% CI, 0.80–0.85) in smaller and larger units, respectively (12, 15). These variable performances are expected when the model is developed using a different population, disease categories, and healthcare systems, including differences in PICU advancement and quality of care provided.

The varying accuracies of predictive models can result from different patient characteristics; the PIM III was initially developed on data gathered from the United Kingdom, Ireland, Australia, and New Zealand Pediatric Intensive Care registries. Of the patients included in their study, 39.7% were admitted for postprocedural recovery compared with 28.8% in our study (6). Moreover, the proportion of the variety of diseases between our study and the original study can influence the accuracy of such models. In particular, metabolic/genetic and immunological diseases can vary widely in type and prevalence between countries, especially the particularities of the Arabian population (21). In the study by Straney et al., 5% of patients were admitted with metabolic or genetic diseases (6). In comparison, 22.6% of our study population had metabolic/genetic comorbidities as a reason for PICU admission. Both models showed different calibration and predictive abilities in the analysis performed on subgroups stratified by disease at admission.

In both models, predicted mortality was lower than observed mortality, as reflected by an SMR of 2.61 and 2.75 for the PRISM III and PIM III predictions, respectively. However, after excluding patients with DNR status from the analysis, the calculated SMR improved to 1.52 and 1.64, respectively. The difference demonstrated in the SMR before and after excluding DNR patients from the calculation is probably related to the difference in the practice of life-sustaining treatments and timing and rationale for withholding specific aggressive support modalities and labeling patients as DNR among different intensivists and institutions. Few studies have examined and demonstrated, without doubt, the impact of culture, religion, and sometimes lack of knowledge on delaying DNR decisions and active withdrawal of support in our community (22–24).

The practice of DNR can differ significantly from one center and culture to another. Determining the DNR status early in the course of an extremely critical patient with a high probability of death is completely different compared with deciding on life support based on quality of life and expected comorbidities after the acute life-threatening presentation is over. Our DNR patients had a significantly longer stay in the PICU, possibly secondary to the slow weaning of support and lack of active withdrawal of life-sustaining management practices in our PICU. It was interesting that 81 of the 222 total mortalities were for cases labeled as DNR and expired inside the PICU after the first week of admission. We are extremely skeptical about the capability of the two-scoring system to predict mortality accurately in this large group.

A sub-analysis of non-survivor patients showed significantly higher PRISM III and PIM III probabilities of mortality in patients who died within the first week of admission. Similar findings were demonstrated by Jacob et al., who demonstrated that these scores performed better at predicting early rather than late mortality (14). DNR patients who died after 1 week had more respiratory and neurological illnesses.

Generally, indices that predict the risk of mortality differ based on patients’ characteristics, medical resources where they were developed, and other factors. The difference in the performance of the PIM III and PRISM III indices in this tertiary PICU compared with that of other health care facilities can be, as demonstrated before, denoted by multiple factors, including the difference in population, disease group, and DNR practice in this institute. DNR patients in this study population had a prolonged length of stay, making the PIM III and PRISM III mortality predictions less accurate. These models should be validated locally before they are implemented as tools for predicting outcomes and measuring performance.

Our study had limitations; it was conducted retrospectively in a single tertiary care center dealing with high-risk patients with multiple comorbidities and fewer trauma patients. In addition, a large number of cases were excluded because of missing data. Additionally, including different ICUs with patients with different disease characteristics may demonstrate different performances for the models tested in this study.

## Conclusion

Both models showed adequate discrimination ability, but poor calibration. We suggest calibrating these models before they are used as quality measures. These models were designed to fit specific patient characteristics and PICUs. Testing these models further in different regional institutions or developing new models to better suit patients admitted to local PICUs is warranted before utilizing them for planning and assessing performance.

## Data Availability Statement

The raw data supporting the conclusions of this article will be made available by the authors, without undue reservation.

## Author Contributions

ASA organized the database, performed the statistical analysis, and wrote the first draft of the manuscript. All authors contributed to the conception and design of the study, wrote sections of the manuscript, and contributed to manuscript revision, read, and approved the submitted version.

## Author Disclaimer

VPS data was provided by Virtual Pediatric Systems, LLC. No endorsement or editorial restriction of the interpretation of these data or opinions of the authors has been implied or stated.

## Conflict of Interest

The authors declare that the research was conducted in the absence of any commercial or financial relationships that could be construed as a potential conflict of interest.

## Publisher’s Note

All claims expressed in this article are solely those of the authors and do not necessarily represent those of their affiliated organizations, or those of the publisher, the editors and the reviewers. Any product that may be evaluated in this article, or claim that may be made by its manufacturer, is not guaranteed or endorsed by the publisher.

## Footnotes

1
http://www.myvps.org

## Abbreviations

AUC-ROC: area under the receiver operating characteristic curve
CI: confidence interval
DNR: do-not-resuscitate
KFMC: King Fahad Medical City
PIM III: Pediatric Index of Mortality III
PICU: pediatric intensive care unit
PRISM III: Pediatric Risk of Mortality III
SMR: standardized mortality ratio.
